# Assessment of image reconstruction algorithm coupled with fine-resolution array of Cherenkov detectors

**DOI:** 10.1038/s41598-022-08158-4

**Published:** 2022-03-12

**Authors:** Luke Maloney, Mackenzie Duce, Anna Erickson

**Affiliations:** 1 University of Florida, Radiation Oncology, Gainesville, FL 32608 USA; 2 Georgia Institute of Technology, Nuclear and Radiological Engineering, Atlanta, GA 30313 USA

**Keywords:** Characterization and analytical techniques, Imaging techniques, Imaging and sensing

## Abstract

The ability to reconstruct fine-resolution images in a high-count-rate environment is an ongoing challenge to the fields of nuclear security, medicine, and high energy physics. This study presents the characterization and performance of an image reconstruction algorithm and detector array in such an environment. The detector array is composed of quartz Cherenkov radiators and lutetium–yttrium oxyorthosilicate inorganic scintillators detector elements with light collection via silicon photomultipliers (SiPM). The reconstruction algorithm was evaluated using ANSI testing standard N42.46-2008 for imaging performance of active interrogation systems for national security applications; this included spatial resolution, wire detection, and penetration studies. The array was tested using a 6-MVp pulsed photon beam where test objects were translated through the detector field of view demonstrating a capability to resolve a 2.05-mm wire at a source standoff of 2.2 m, a horizontal spatial resolution of 3 mm, and a contrast sensitivity of 1.5%.

## Introduction

Demand for fine-resolution imaging in high-dose applications, including high-energy physics, medical physics, and nuclear security place a number of requirements on detection systems: withstand potential radiation damage, operate with short decay time, and function with high background present^1,2^. Typically, scintillators are used in imaging applications because of their energy resolution capabilities, especially in active interrogation space^3,4^. However, even fast scintillators, like LYSO, can become saturated in high-rate environments due to their long decay time (tens of nanoseconds)^5^.

In comparison, Cherenkov radiators present a viable alternative as a photoconverter for spatial imaging applications that are low in cost^6^, capable of low-energy background rejection, and have fast signal processing^2^. Further, Cherenkov radiators have recently been demonstrated to be applicable for material discrimination (or $Z_{eff}$) imaging, as shown by Rose and Erickson^2^. In medical physics, Cherenkov detectors have been shown to have imaging capabilities in applications where skin tissue is used as the scattering medium^7^. In volcanology, Cherenkov detectors have been proposed as a method for retrieving information on a volcano’s activity^8^. Recently, Cherenkov detectors have been explored for use in active interrogation for national nuclear security applications^2,9^.

The lack of energy resolution associated with Cherenkov detectors impedes fine-resolution imaging in certain applications, when various energies of gamma-rays are used to extract information about the object, but can be improved with careful image reconstruction. In this work, we evaluate the performance of an novel image reconstruction algorithm with a hybrid Cherenkov-scintillator imaging array system with light collection via silicon photomultipliers (SiPM). SiPMs are an emerging, favorable alternative to traditional photomulitplier tubes (PMT)^10^. One notable challenge to image reconstruction due to SiPMs discussed here is the asymptotic behavior of their saturation in high-flux environments, for which a mathematical model is proposed. Additionally, discussion of time- and energy- dependent features of the data relevant to image reconstruction process is included for use in future works involving Cherenkov-SiPM photocouples. The array, consisting of both LYSO and Cherenkov radiators, utilizes the good energy resolution of inorganic scintillators with the fast timing of Cherenkov radiators. The imaging performance of the array and novel algorithm were evaluated using the ANSI standard for imaging performance of X-ray and gamma-ray systems for cargo and vehicle security screening, ANSI N42.46-2008^11^.

## Methods

### Experiment design

The system described here consists of an array of both Cherenkov radiator and scintillator detector elements with light collection via silicon photomultipliers (SiPM) on a custom printed circuit board (PCB). Data acquisition was conducted with two CAEN DT5730 digitizers with image reconstruction performed by a custom algorithm described in this paper. For the results shown, test objects were translated across the beam of a clinical-grade linear accelerator.

**Figure 1:**
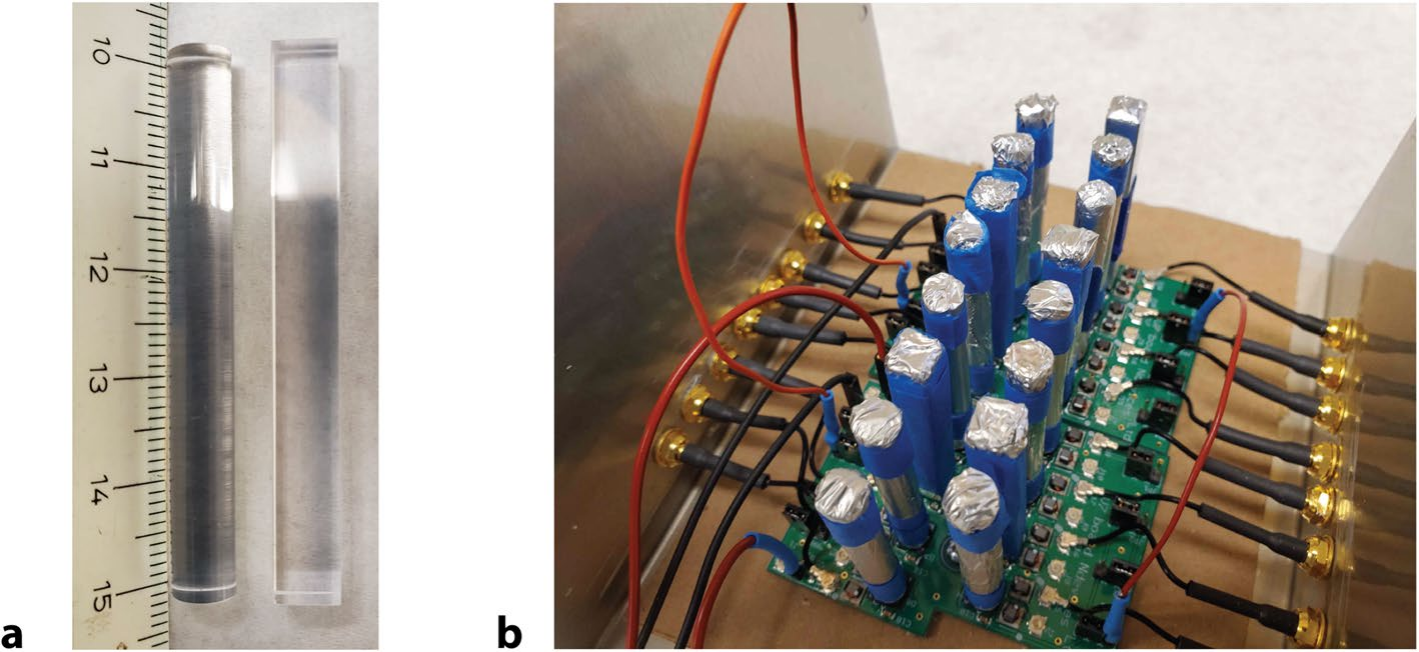
(**a**) Right: LYSO detector volume element; left: quartz volume element. (**b**) Assembled array, with top of lightproof enclosure removed. Orange and red cables daisy chain the bias voltage between boards. The inner portion of the MCX bulkheads is visible.

The inorganic scintillator was LYSO (Scintitech, Shirley, MA, USA), selected for its fast light output and efficiency. Quartz rods, sourced from Technical Glass Products (Painesville, OH, USA), were employed here as Cherenkov radiators. Both detector elements have dimensions of 6 mm diameter by 50 mm length geometry, matching the 6-mm × 6-mm cross-sectional geometry of the SiPM windows. The LYSO and quartz elements are seen in Fig. 1 along with the assembled array.

To maximize light collection, detector volumes were lightproofed by double wrapping in reflective material: an inner layer of polytetrafluoroethylene (PTFE) tape and an outer layer of aluminum foil. Optical coupling to the SiPM windows, sourced from ON Semiconductor (Cork, Ireland), was accomplished by use of RX-22P silicon coupling compound (Rexon Components, Beachwood, OH)^12^. The custom PCB, seen in Fig. 1, was designed to minimize cross-talk among detector elements and thus has a unique geometry^13^.

The imaging source used was a Clinac linear accelerator (Varian Medical Systems, Palo Alto, CA, USA), located at the Georgia Institute of Technology. The device can produce bremsstrahlung photon beams of ${6}\,\hbox { MeV}$ and ${18}\,\hbox { MeV}$ endpoints. As the device uses microwave-driven acceleration within a standing waveguide, the beam is pulsed at a rate of ${2.78}\,\hbox { ms}$ per pulse, or ${360}\,\hbox { Hz}$. The instantaneous (per-pulse) dose rate in this device is not guaranteed to be constant—the system described here is agnostic of the pulse magnitude, permitting imaging with a priori knowledge only of the total beam fluence over the duration of the acquisition time. Additional factors necessary for reconstruction are derived from this and other measurements described in detail later.

Imaged objects were translated across the system field of view (FOV) by a 100-mm linear translation stage (Thorlabs, Newton, NJ, USA), driven by a BSC20x stepper motor controller. For all acquisitions, maximum allowed acceleration was ${1}\,{\hbox { mm}/\hbox {s}^{2}}$ and velocity was ${1}\,{\hbox { mm}/\hbox {s}}$, unless otherwise noted. Due to the unique design of the array having two rows of detectors, the effective field of view of the system is less than the travel distance: In order to acquire a complete object scan, the object must traverse a distance *d* equal to

$$d = w_d + p \, ,$$

where $w_d$ is the detector width in the direction of travel and *p* the pitch in the same direction. Thus, each image presented whose dimension in the direction of travel exceeds the FOV distance is a concatenation of two or more individual acquisitions.

### Image reconstruction

**Figure 2:**
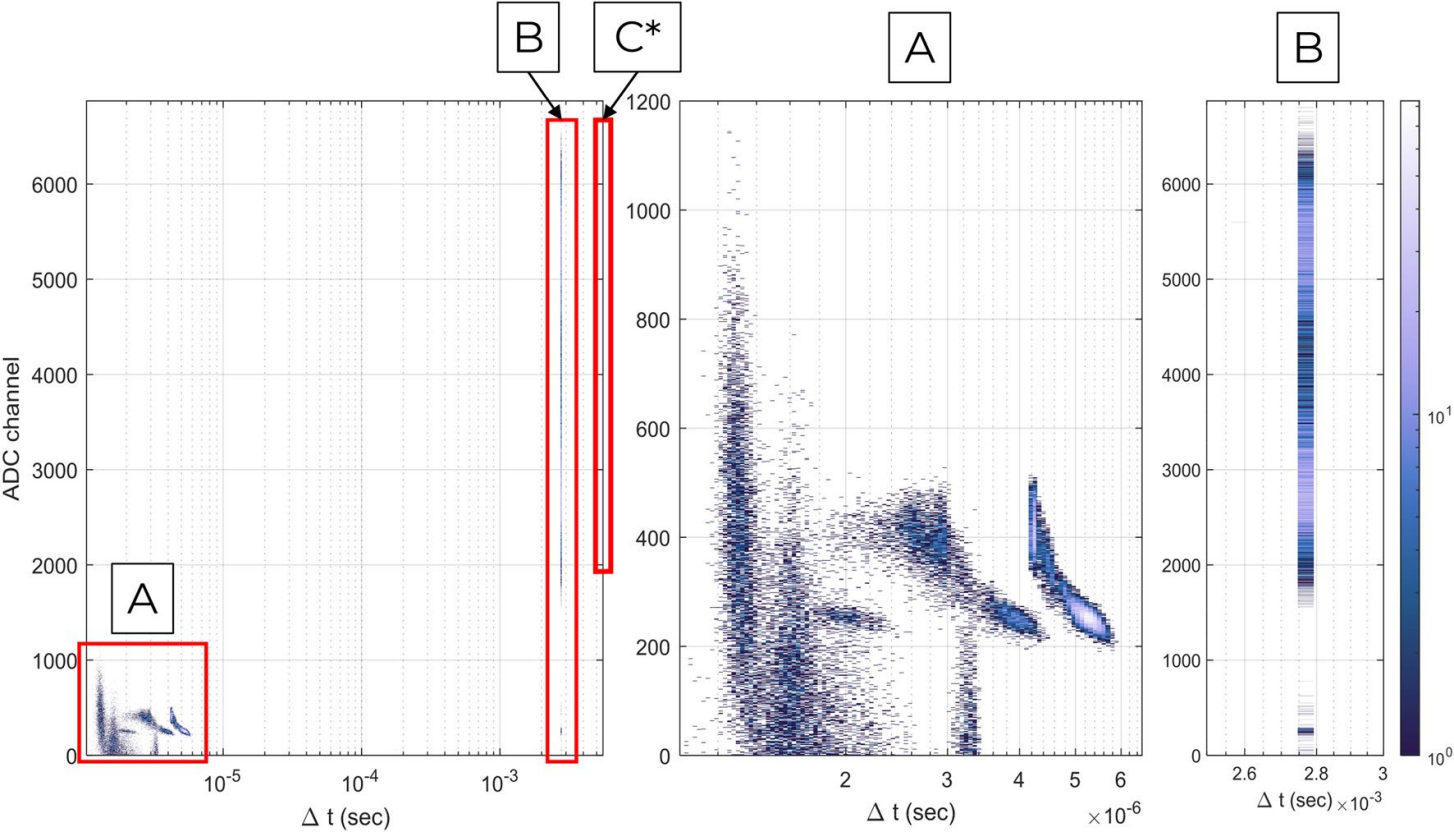
Correlated noise and signal counts in quartz detector, shown as a bivariate histogram of difference in time tag between successive events and recorded event signal, in ADC channels, for a single quartz photocouple (index 6). Bin edges are logarithmically spaced in $\Delta$t, and linearly in ADC (${501 \times 16384}$ edges). Detail views are shown for regions A and B. Region C contains only 60 counts in the rightmost $\Delta$t bin, [5.5,5.6] ms.

Cherenkov detectors are typically not used in imaging applications because of their inefficient conversion of electron kinetic energy to optical photons for collection by PMT’s or analogous devices. When a high-intensity pulsed source is used, the single-pulse signal level is sufficiently large to enable ADC light readout for spatial imaging. As shown in Fig. 2, the inter-time histogram allows clear delineation of signal counts and correlated noise counts, which occur promptly. Figure 2 shows the time since the last event to the next for each event in a single acquisition. This is illustrative of the actual source of signal in the device. As mentioned previously, the imaging source is pulsed at ${2.78}\,\hbox { ms}$ per pulse, thus the signal counts are those in the rightmost regions B and C. Region C contains counts that are due to “dropped” pulses in the linear accelerator pulse train, which occasionally occur in order to maintain a constant dose rate for its typical application as a medical device.

The region A in Fig. 2 bears particular mention because of the relative frequency and distribution of the counts. As shown in previous work^14,15^, these events are prompt noise occurring strictly within the SiPM. Some prompt noise events are due to afterpulsing, which occurs due to electrons crossing the p-n junction, becoming trapped, and subsequently released from metastable traps. Others arise from “cross-talk” among single-photon avalanche diode (SPAD) sites. Infrared photons may be released during the primary interaction of an optical photon with the silicon substrate and subsequently reabsorbed in the matrix. Prompt noise has been discussed in the literature^14–17^, but can be readily discriminated against on the basis of time-to-event when a pulsed source is in use. If necessary, ADC windowing can also be used, since the prompt noise events are almost always lower in energy than signal counts, though some signal counts will erroneously be windowed out in this approach, particularly if available imaging flux is not sufficiently intense to generate signal in the upper ADC channels.

A major issue when employing scintillators in a similar operational mode as Cherenkov radiators is saturation of the signal level. Though energy resolution as low as 11% has been demonstrated for LYSO-SiPM systems^18^, radiant intensity has a strong effect on system calibration: in an avalanche photodiode (APD) array like a SiPM, the signal level depends on the number of fired microcells. A number of factors influence the firing microcells for a given interaction event. The total number of optical photons generated in the radiator (of either type), which in turn depends on radiator geometry, is the most significant. In high-aspect ratio radiators, electron escapes from the medium may lead to incomplete energy deposition, thus adding further uncertainty to the total number of photons generated. Photon transport in the form of reflection, refraction, and absorption reduces the available signal to be converted to photoelectrons. Thus, the output signal is a nonlinear function of the number of incident photons, that in turn further depends on the photon detection efficiency, microcell recovery time constant, cross-talk between APD elements, and “dark” counts which occur spontaneously even in the absence of incident photons.

While it is difficult to directly compare scintillators to Cherenkov radiators, the common metric of light yield—the number of optical photons generated in the medium per unit energy deposited—serves as a useful heuristic. Typically reported in photons per $\hbox { MeV}$ for scintillators, it is an indicator of the signal-to-noise level expected due to physical processes in the medium. For sodium iodide (NaI(Tl)), one of the most common inorganic scintillators, the light yield is ${38.0}{/\hbox {keV}}$, while for LYSO it is between ${27.6/\hbox {keV}}$ to ${33.2}{/\hbox {keV}}$. In Cherenkov radiators, the number of photons available to be collected at the APD is very low—a consequence of the poor efficiency of the light production; the number of photons produced follows the Frank–Tamm formula, Supplementary Equation 1. The cross section increases (in an affine sense) as the square of the particle velocity, though emission is strongly peaked in the ultraviolet, as shown in Supplemental Fig. 1. By integration over wavelength and accounting for electron stopping power, the light yield in the megavoltage electron energy range is on the order of 100 to 300 photons per $\hbox { MeV}$ of deposited energy, as shown in Supplemental Fig. 2, orders of magnitude below the optical signal level expected in a scintillator medium.

For spatial imaging applications, an intuitive mapping of signal to the image domain has the signal level proportional to the number of incident photons. If a random photon has equal probability of striking any given microcell in a detector, the probability that *U* unfired microcells exist in a SiPM consisting of $N_{cells}$ in total is a binomial distribution as detailed in Supplemental Information. When the number of incident photons is small relative to the number of available microcells, the curve is nearly linear, as shown in Supplemental Fig. 3. In order to linearize the image level in the processed image, we apply a pixel-wise calibration with image transmission ratios mapped to the theoretical ratios given by the 1-dimensional Beer-Lambert attenuation (the argument of the logarithm is squared to ensure positivity).

**Figure 3:**
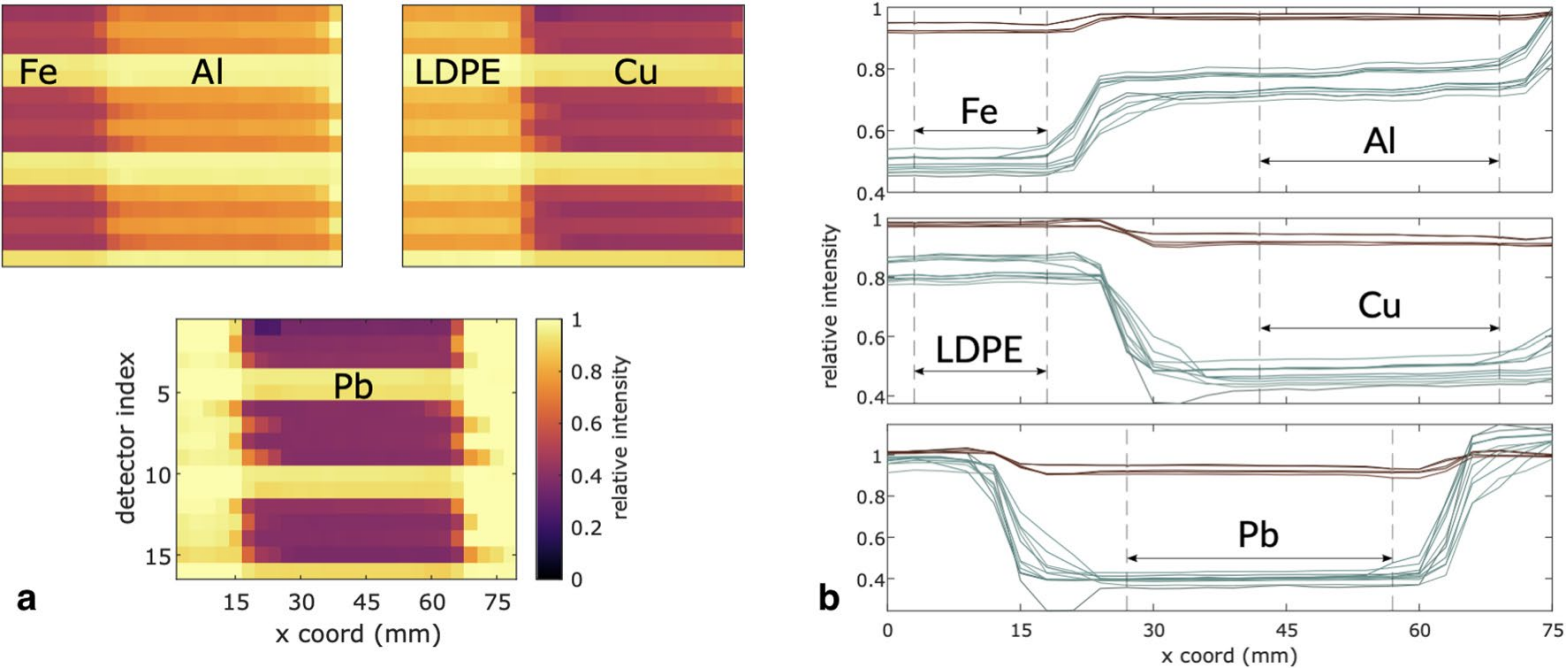
Material images forming basis for cross-calibration of array channel responses. (**a**) Top left: iron and aluminum blocks abutting in a reconstructed image; Top right: LDPE and copper blocks abutting; Bottom: lead block centered on a bright field. (**b**) Profiles across material images as basis for response matching. Cherenkov channel profiles shown in blue and form the lower band of 11 profiles, while the upper band of profiles with less contrast correspond to LYSO response. Regions of interest for each block are indicated.

Calibration images that form the basis for the linearizing fit are shown in Fig. 3a. Single-acquisition images were acquired for solid blocks of known thickness of several materials: iron (Fe), aluminum (Al), copper (Cu), low-density polyethylene (LDPE), and lead (Pb). From the uncorrected block profiles of image intensity, shown in Fig. 3b, for each detector channel, regions of interest (ROI) were selected in which the slope was nearly constant. Spatially averaged raw intensities were fit (in a nonlinear-least squares sense) to the data by Supplemental Equation 5. The fit parameters and residuals for calibration curve fits can be found in Supplemental Table 1 and Supplemental Fig. 4, respectively. Scattering effects are assumed to be very small with respect to the image signal, primarily because the detective solid angle subtended, even by the entire array, at the object plane is small with respect to the expected distribution of photon trajectories originating within the object. Inter-detector scatter for a closely related system has been modeled and benchmarked against experimental results concluding that cross-talk between channels contributes less than 1% of counts for quartz detectors of 50-mm diameter with 5-mm spacing in^13^. The system used here features larger spacing and smaller diameter quartz detectors, so cross-talk is assumed to be negligible.

## Imaging results

The measurement setup and test conditions for the imaging system employed a Varian Clinac iX linear accelerator at the Radiological Science and Engineering Laboratory, Georgia Institute of Technology. Because the system was developed for cargo imaging applications, imaging performance was evaluated based on metrics modified from ANSI N42.46-2008^11^.

### Wire detection

**Figure 4:**
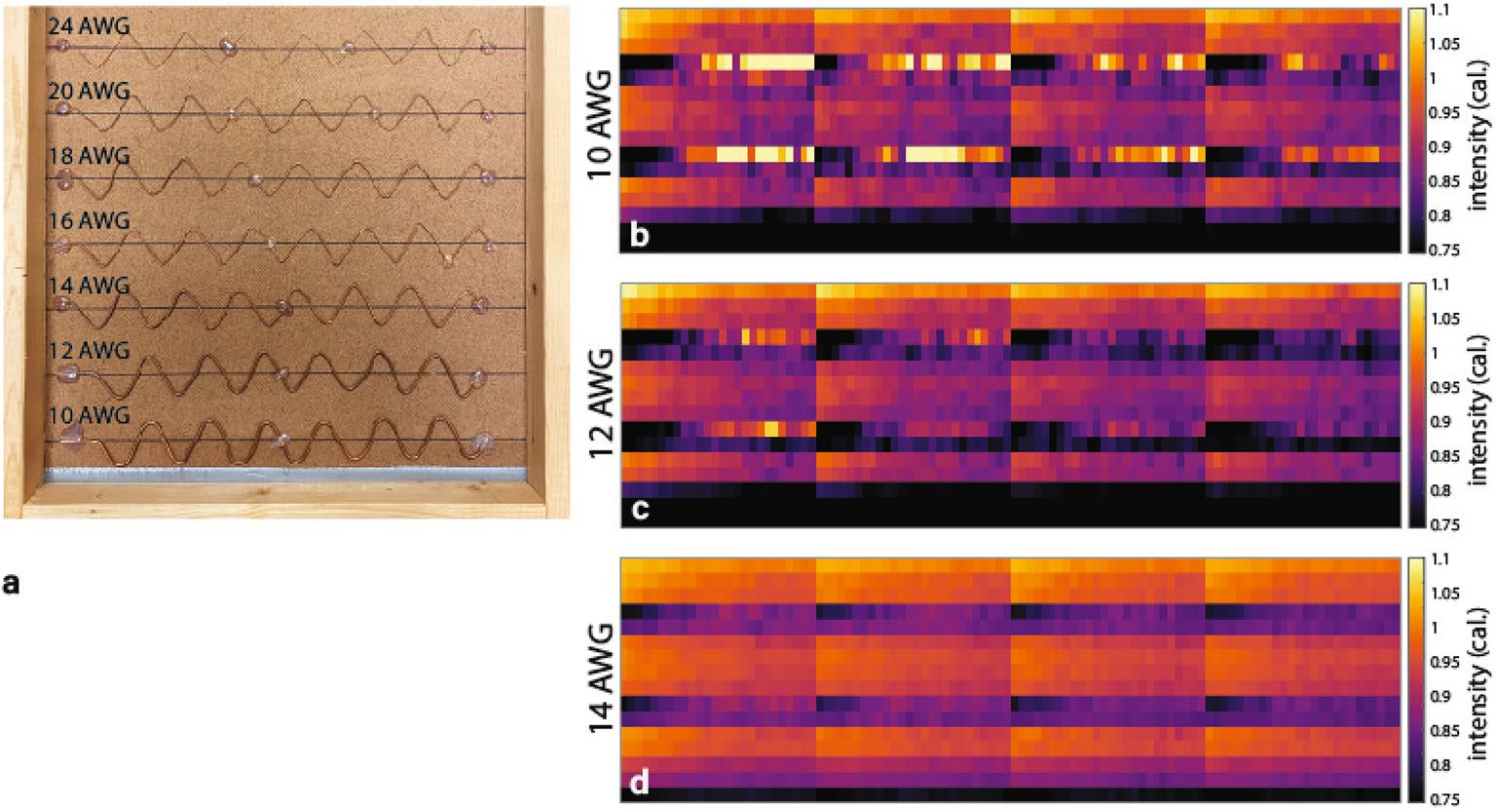
(**a**) Images of wire detection test object, consisting of bare copper wire of varying gauge. (**b**-**d**) ANSI N42.46-2008 wire sensitivity test results, for three thickest wires in object. All images were calibrated using the method described above. Bright patches in LYSO channels are due to large differential in calibrated image level with respect to SiPM response—i.e. the steep slope of the calibration curve for LYSO. Images were re-windowed to facilitate ease of reading, see color bars at right. (**b**) Wire sensitivity test, 10-AWG wire. (**c**) Wire sensitivity test, 12-AWG wire. (**d**) Wire sensitivity test, 14-AWG wire.

The test object for wire detection consisted of bare copper wire bent into sinusoids, each at least four periods in length and of 25-mm peak-to-trough amplitude, and mounted to a thin chipboard backing, as shown in Fig. 4a. Wire detection test images, shown in Fig. 4b–d, have immediately evident artifacts: there is streaking and speckling across the LYSO profiles, as well as drop in image intensity from right to left. These result from several factors. First, the image acquisition, beam-on time, and stepper motor initiation are asynchronous, so any beam intensity degradation near the start of acquisition manifests as a drop in image intensity from right to left. There exists a balance which must be struck between using high enough flux to achieve appreciable Cherenkov signal and low enough such that the LYSO signal’s energy resolving capability is usable without saturating the SiPM. In particular, concatenation of subsequent acquisitions manifests as vertical streaks separated by the length of the FOV. Second, speckling arises from the instability of the calibration of primarily the LYSO channels. The dark streaks near the bottom of each image are a consequence of the calibration method. During the experiment, the material blocks were placed directly on the stage which is absent from the wire images; therefore, pixel values before calibration were artificially low, and the transmission ratio was underestimated.

The 10-AWG wire (diameter of ${2.588}\hbox { mm}$) object is easily discernible, though the image features which permit correct identification come primarily from the Cherenkov channels. The image banding is darker in the case of the 12-AWG (diameter of ${2.053}\hbox { mm}$) image, but the wire may still be positively identified as present in the acquisition. The 14-AWG object (diameter of ${1.628}\hbox { mm}$) is difficult if not impossible to identify in the image; consequently, smaller diameter wire object images are not shown here.

### Line pair resolution

The ANSI standard imaging test specifies that the test object consist of a steel line-pair gauge, with either air slots in a steel plate or three steel rods in air, which have width and thickness of *d*, are separated by a distance *d*, and are ${100}\hbox { mm}$ long. In this test, nails were used as steel rods (with circular rather than square cross-section), as described in Table 1.

**Table 1:** Diameters of objects used in spatial resolution test.

Nail size	Length (mm)	Diameter (mm)
3D	31.8	1.87
6D	50.88	2.90
16D	88.9	4.14
20D	102	4.97

Vertical spatial resolution is difficult to determine primarily as a result of time-dependent response degradation, as described in the previous section. The degradation cannot be accurately corrected for in a time-dependent normalization due to non-concurrency of initiation of system acquisition components: stage, accelerator, and DAQ. Consequently, image striping is partly uncorrected, and vertical spatial resolution is not discussed here.

**Figure 5:**
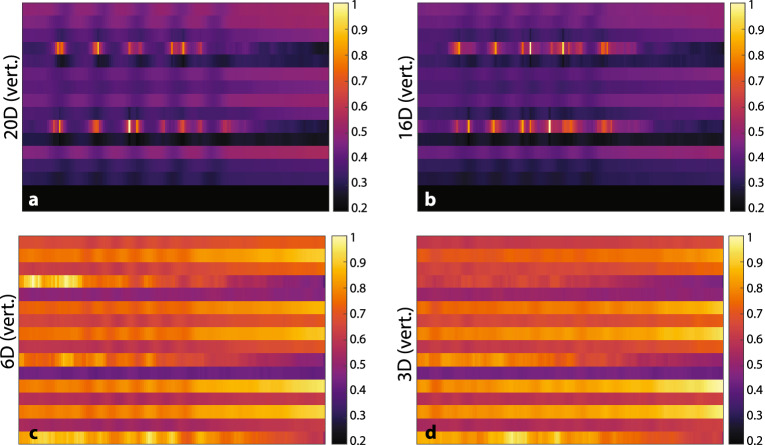
Horizontal spatial resolution test result images. (**a**) 20D nails, vertical orientation. (**b**) 16D nails, vertical orientation. (**c**) 6D nails, vertical orientation. (**d**) 3D nails, vertical orientation. The line pair gauges are easy to discern for the thicker gauges, and are only not detectable for the smallest test objects imaged.

Horizontal spatial resolution results show that line pairs can be easily distinguished even for objects having $d={2.90}\hbox { mm}$, as seen in Fig. 5. The 20D, 16D, and 6D test objects are all identifiable as line pairs.

**Figure 6:**
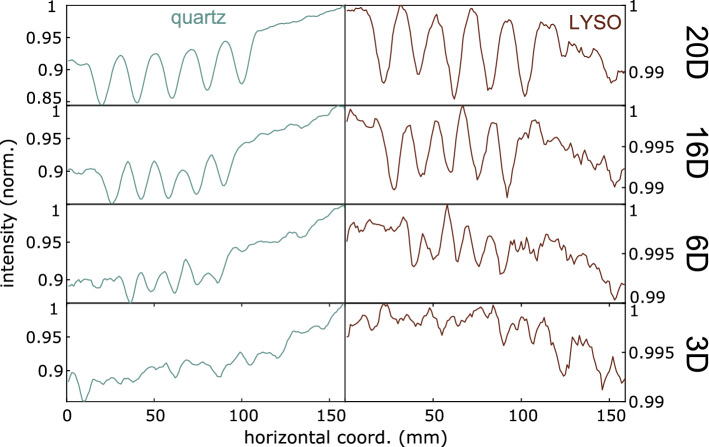
Horizontal spatial resolution image profiles, corresponding to images in Fig. 5. Note the large difference in dynamic range between LYSO and quartz peaks. (**a**) 20D nails, horizontal spatial profiles. (**b**) 16D nails, horizontal spatial profiles. (**c**) 6D nails, horizontal spatial profiles. (**d**) 3D nails, horizontal spatial profiles. Horizontal spatial resolution image profiles, corresponding to images in 5, in detector indices 6 (quartz) and 11 (LYSO). Effect of LYSO instability of calibration on noise is evident in those profiles.

The horizontal profiles corresponding to Fig. 5 are shown in Fig. 6. The “baseline” intensity to which profiles return between objects is nonuniform due to apparent output decrease during acquisition, but the imaging results are corroborated: The number of valleys in the 20D and 16D plots is clearly that expected from the number of line pairs, though in the 16D case the interobject peak height has greater variation. For LYSO profiles in each case, there are less prominent peaks within the valleys, which arise due to aforementioned calibration instability. In the 6D case, the careful observer might identify the correct number of valleys, especially for the quartz profile, which is impractical in the case of the 3D object, due to the profile having no easily distinguishable peaks.

### Penetration and contrast sensitivity

**Figure 7:**
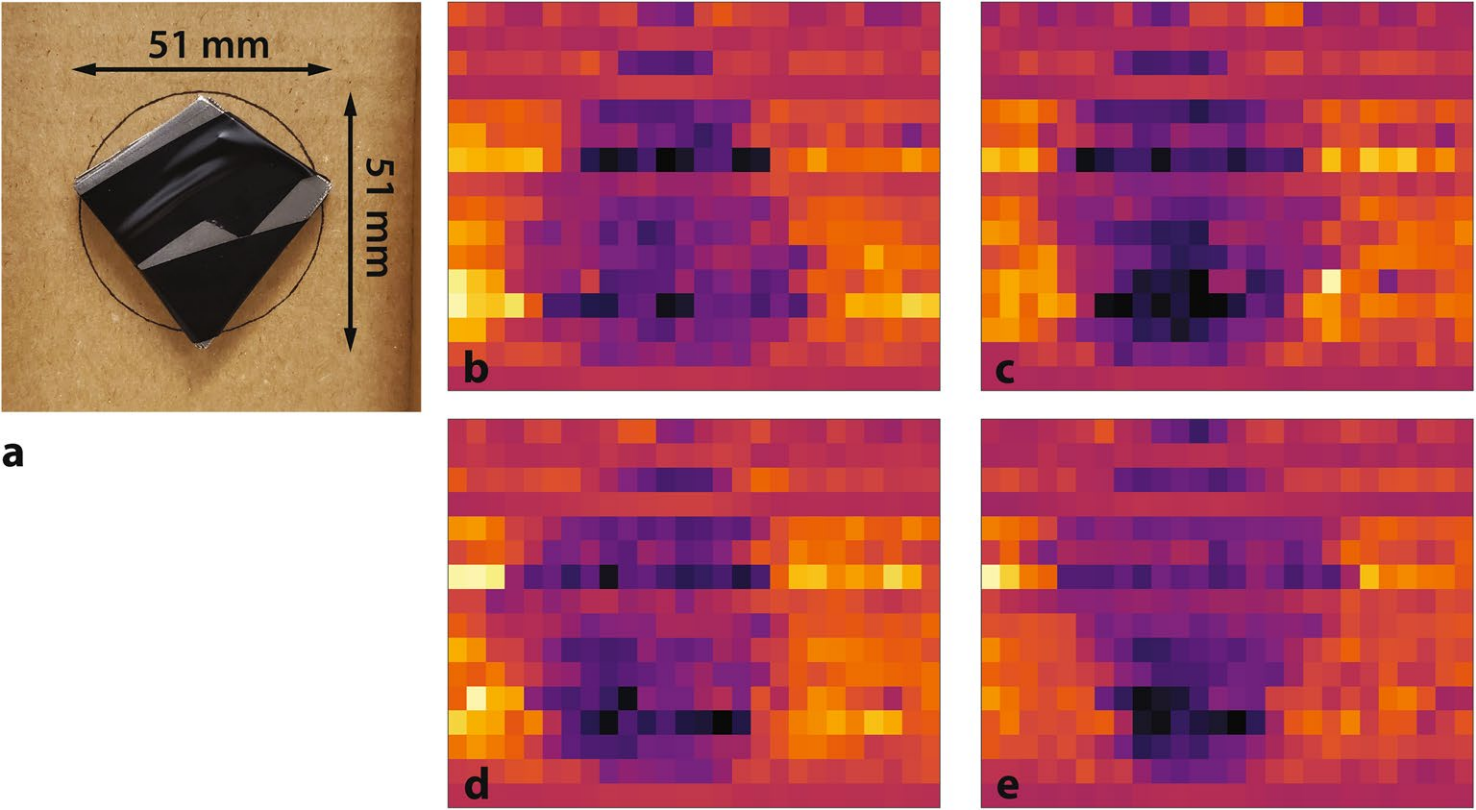
Test object blocked by ${102}\hbox { mm}$ of lead. The object is distorted such that it is compressed in the lateral dimension, due to the pixel size of the image (${2.4 \times 3}\hbox { mm}$). (**a**) Arrow test object. (**b**) 102 mm lead blocking, arrow up. (**c**) 102 mm lead blocking, arrow right. (**d**) 102 mm lead blocking, arrow left. (**e**) 102 mm lead blocking, arrow down.

The ANSI penetration and contrast sensitivity test object “is a steel arrowhead shape 30-cm long and 30-cm wide”^11^. The prototype array is significantly smaller than these dimensions—the scaled test object is a steel arrowhead seen in Fig. 7a, consisting of nine 22-gauge sheet metal shims (thickness of ${0.759}\hbox { mm}$ each). Contrast sensitivity was measured by varying the thickness of the test object—by changing the number of shims—and the thickness of the blocking plates while the arrow is rotated in a random direction, and the observer attempts to correctly identify the direction the arrow points. The N42.46-2008 standard specifies that steel blocking plates be used; here, steel-equivalent thicknesses of lead are reported.

In a contrast sensitivity test wherein the test object at full thickness (of ${6.83}\hbox { mm}$) is blocked by ${102}\hbox { mm}$ of lead (equivalent to ${150}\hbox { mm}$ of steel at an effective beam energy of ${2}\hbox { MeV}$), as in Fig. 7, the orientation of the arrow can be readily discerned in each case, despite image artifacts as described above. Here, penetration through the test object is 19%, while penetration through the lead blocking plates is 0.04%, corresponding to a contrast sensitivity

1 $$\begin{aligned} CS = \frac{t_{arrow}}{t_{plates}} \end{aligned}$$

of 6.7%.

**Figure 8:**
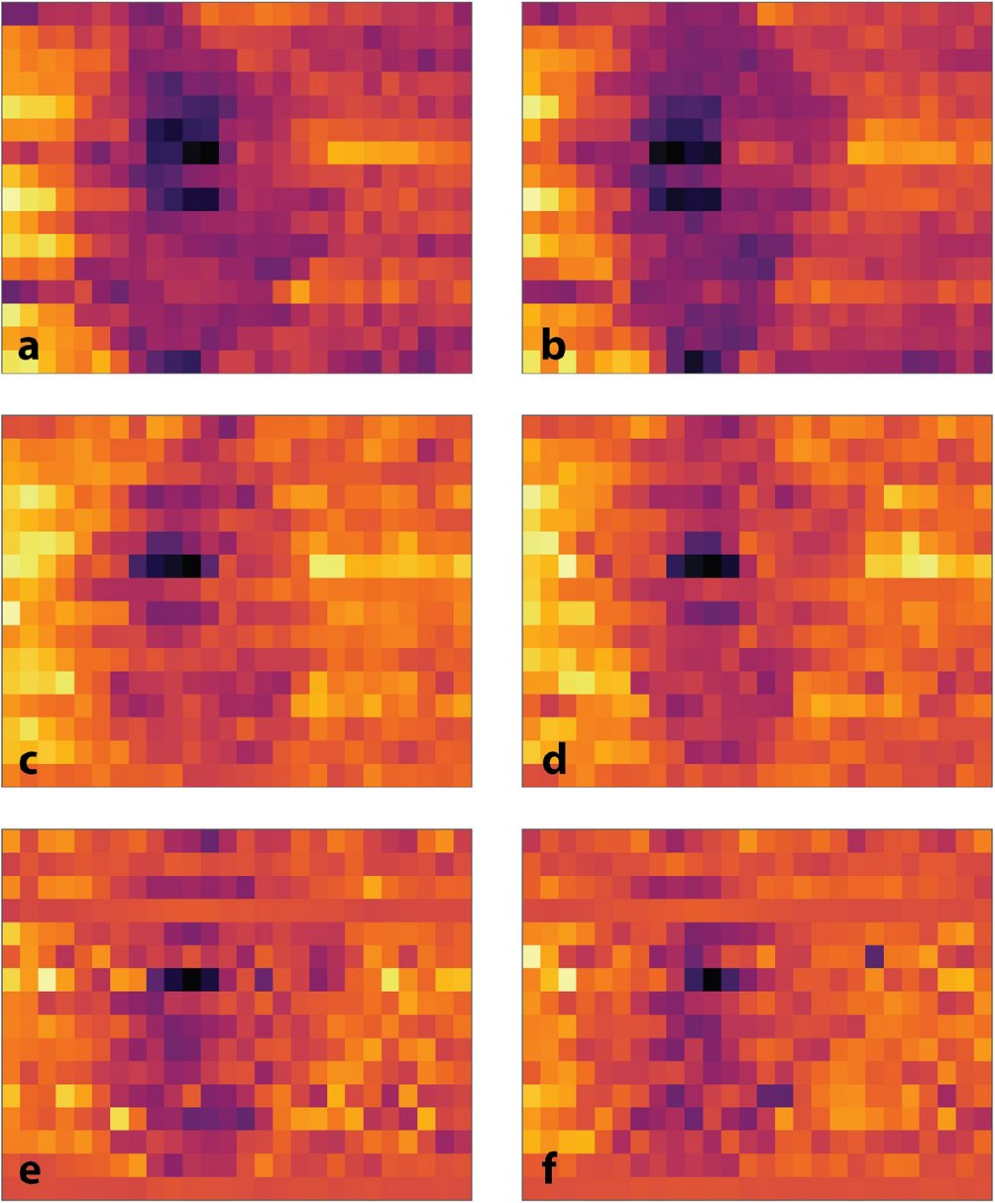
Contrast sensitivity test at one third thickness. The object is distorted such that it is compressed in the lateral dimension, due to the pixel size of the image (${2.4 \times 3}\hbox { mm}$). (**a**) 30-mm lead blocking, arrow pointing right. (**b**) 30-mm lead blocking, arrow pointing down. (**c**) 61-mm lead blocking, arrow pointing left. (**d**) 61-mm lead blocking, arrow pointing down. (**e**) 102-mm lead blocking, arrow pointing down. (**f**) 102-mm lead blocking, arrow pointing right.

The contrast sensitivity test is used to measure observer feature identification (in this case, arrow orientation) as object thickness varies with respect to blocking plate thickness. Figure 8 shows the results of the contrast sensitivity test images for an object of one-third the original thickness. It is important to note that this is a subjective test dependent on the reader. Contrast sensitivity values at one-third object thickness are 4.55%, 2.28%, and 1.52% for lead blocking plate thicknesses of $30\;{\text{mm}},\;61\;{\text{mm}}\;{\text{and}}\;102\;{\text{mm}}$, respectively. These values may be used to evaluate the images in Fig. 8.

## Conclusion

This work shows that in a high-flux, pulsed source application space, quartz is a viable (and possibly preferable) alternative to dense scintillators due to its resistance to over-saturation effects resulting from SiPM readout. The system is sufficiently sensitive to detect 2.05-mm wire at a standoff distance of ${2.2}\hbox { m}$ source-to-object and ${4.6}\hbox { m}$ source-to-detector. Horizontal spatial resolution is better than ${3}\hbox { mm}$, and contrast sensitivity may be as low as 1.5%, though this subjective metric depends on the reader of the study. This system demonstrates that the high-rate environment necessary for active interrogation is not a limiting factor in achieving satisfactory imaging of the target, even with some shielding present, with the use of Cherenkov detectors and proper image reconstruction.

## Supplementary Information

Supplementary Information.

## Data Availability

Data available upon request.
