# Supplementary material for: Assessment of image reconstruction algorithm coupled with fine-resolution array of Cherenkov detectors
Source: Sci Rep. 2022 Mar 12;12:4311. doi: 10.1038/s41598-022-08158-4 (PMC8918355; doi:10.1038/s41598-022-08158-4)
Supplement: Supplementary file 1 — Supplementary Information. [file 41598_2022_8158_MOESM1_ESM.pdf]

Supplementary Information

The differential cross section for photon generation by electrons in dense media is given by the Frank-Tamm formula:

$$\frac{d^2N}{dx\,d\lambda} = \frac{2\pi\alpha}{\lambda^2}\left(1 - \frac{1}{\beta^2 n^2}\right), \tag{1}$$

where α is the fine structure constant, n is a function of λ, the photon wavelength, and $\mu \approx \mu_0$, assuming medium permeability and vacuum permeability are approximately equivalent.

While it is difficult to directly compare scintillators to Cherenkov radiators, the common metric of light yield—the number of optical photons generated in the medium per unit energy deposited—serves as a useful heuristic. Typically reported in photons per MeV for scintillators, it is an indicator of the signal-to-noise level expected due to physical processes in the medium. For sodium iodide (NaI(Tl)), one of the most common inorganic scintillators, the light yield is 38.0/keV, while for LYSO it is between 27.6/keV to 33.2/keV. In the Cherenkov processes producing light in media, the number of photons follows Equation 1. The cross section increases (in an affine sense) as the square of the particle velocity, though emission is strongly peaked in the ultraviolet, as shown in Supplementary Fig. 1. By integration over wavelength and accounting for electron stopping power, the light yield per unit kinetic energy as an electron stops is given by

$$LY\left(T_0\right) = \frac{1}{\rho\, T_0}\int_0^{T_0}\int_\lambda \left(\frac{d^2N}{dx\,d\lambda}\right)\left(\frac{dT}{\rho\, dx}\right)^{-1} d\lambda\, dT\,, \tag{2}$$

where T_0 is the initial kinetic energy of photoelectrons in the radiator medium. The light yield in the megavoltage electron range is on the order of 100 to 300 photons per MeV of deposited energy, as shown in Supplementary Fig. 2, orders of magnitude below the optical signal level expected in a scintillator medium.

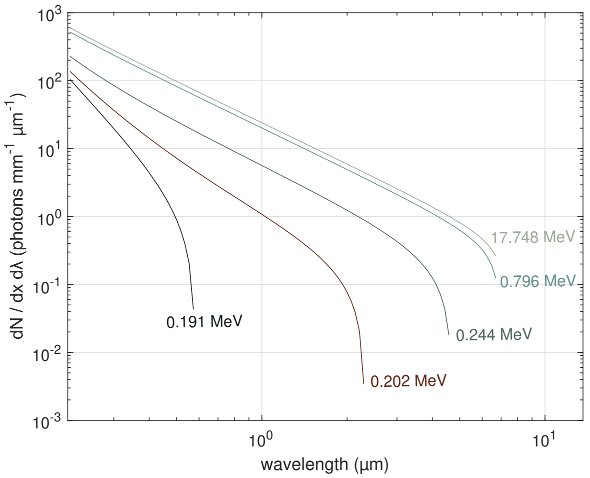

Supplementary Figure 1. Cherenkov spectra in quartz. These curves correspond to electrons in material following backscatters from photons of 0.336 MeV, 0.350 MeV, 0.4 MeV, 1 MeV and 18 MeV.

For spatial imaging applications, an intuitive mapping of signal to the image domain has the signal level proportional to the number of incident photons. If a random photon has equal probability of striking any given microcell in a detector, the probability that U unfired microcells exist in a SiPM consisting of N_{cells} in total is

$$p(N_{\text{cells}}, N_\gamma) = \binom{N_{\text{cells}}}{U}\sum_{v=0}^{N_{\text{cells}}-U}(-1)^v\binom{N_{\text{cells}}-U}{v}\left(1 - \frac{U+v}{N_{\text{cells}}}\right)^{N_\gamma} \tag{3}$$

for a given time of exposure, and ignoring PDE (see, for example,[1]).

An exponential model that closely follows this binomial distribution[2,3] is

$$N_{\text{fired}} = N_{\text{cells}}\cdot\left(1 - e^{-\frac{N_\gamma\cdot\text{PDE}}{N_{\text{cells}}}}\right), \tag{4}$$

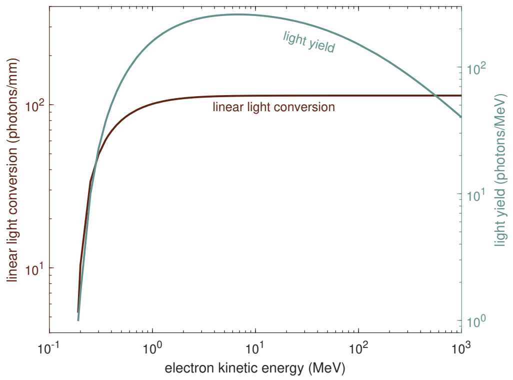

Supplementary Figure 2. Linear light output and total per-MeV light yield in quartz as a function of electron kinetic energy. The peak near 6 MeV-7 MeV is due to the minimum in the stopping power near this region. NIST values for plate glass were used in place of quartz.

as shown in Supplementary Fig. 3. When the number of incident photons is small relative to the number of available microcells, the curve is nearly linear. In order to linearize the image level in the processed image, a pixelwise calibration of the form

$$\left(\frac{I}{I_0}\right)_{\text{theoretical}} = a \cdot \log\left(\left(1 - b \cdot \left(\frac{I}{I_0}\right)_{\text{image}}\right)^2\right) + c \tag{5}$$

is applied, with image transmission ratios mapped to the theoretical ratios given by the 1-dimensional Beer-Lambert attenuation (the argument of the logarithm is squared to ensure positivity). Upper bounds of 0 and 1 are used for a and b, respectively, to preserve concavity and satisfy the condition $1/b > 1$, such that the asymptote corresponding to total saturation does not occur on the interval of the image intensity dynamic range.

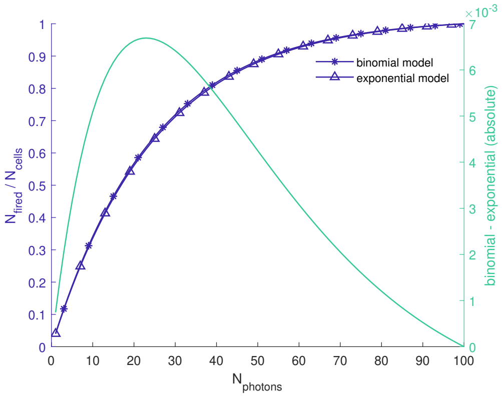

Supplementary Figure 3. Comparison of binomial model (Equation 3) and exponential model (Equation 4) for fraction of fired microcells in a SiPM consisting of 25 microcells total.

Fit parameters for each of the channel calibrations (Cherenkov, "C," or LYSO, "L") are given in Supplementary Table 1, and residual distributions are given in Supplementary Fig. 4. Metrics of adjusted R-square and RMSE are generally poorer for the LYSO channels. LYSO, which is a much more efficient photoconverter with higher light yield than quartz, exhibits a poor conditioning in the calibration, and small changes in input image level correspond to large changes in the output.

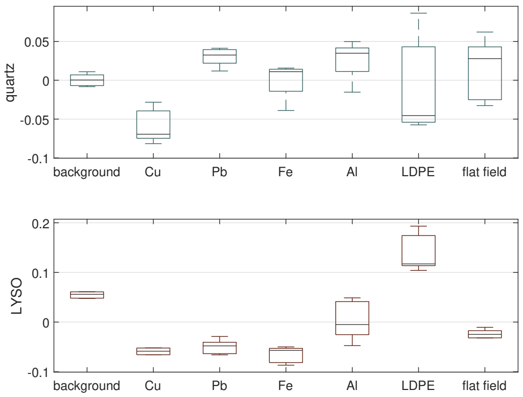

Supplementary Figure 4. Box-and-whisker plot of residuals for calibration curve fits.

Supplementary Table 1. Fit parameters, and goodness of fit

Channel	Type	a	b	c	R^2_{adj}	RMSE
1	C	-3.330	0.145	-0.008	0.9769	0.0538
2	C	-0.668	0.509	0.008	0.9718	0.0594
3	C	-0.755	0.498	-0.011	0.9876	0.0395
4	L	-0.091	0.998	-0.088	0.8916	0.1165
5	L	-0.108	0.995	-0.113	0.9372	0.0887
6	C	-0.727	0.476	-0.003	0.9671	0.0642
7	C	-0.698	0.516	-0.001	0.9915	0.0326
8	C	-0.619	0.536	0.007	0.9719	0.0593
9	C	-0.699	0.500	0.004	0.9764	0.0543
10	L	-0.092	0.998	-0.093	0.9119	0.1051
11	L	-0.109	0.995	-0.106	0.9420	0.0853
12	C	-0.477	0.632	0.006	0.9730	0.0581
13	C	-0.960	0.418	-0.009	0.9885	0.0380
14	C	-0.571	0.563	0.007	0.9735	0.0576
15	C	-0.706	0.511	0.000	0.9931	0.0293
16	L	-0.102	0.996	-0.091	0.9541	0.0758

References

1. Feller, W. *An Introduction to Probability Theory and Its Applications*, vol. 1 (Wiley, 1968).

2. Renker, D. & Lorenz, E. Advances in solid state photon detectors. *J. Instrumentation* **4**, P04004–P04004, DOI: 10.1088/1748-0221/4/04/p04004 (2009).

3. Gruber, L., Brunner, S., Marton, J. & Suzuki, K. Over saturation behavior of sipms at high photon exposure. *Nucl. Instrum. Methods Phys. Res., Sect. A* **737**, 11 – 18, DOI: 10.1016/j.nima.2013.11.013 (2014).
